# Psychobiological Factors in Global Health and Public Health

**DOI:** 10.3390/ijerph19116728

**Published:** 2022-05-31

**Authors:** Frédéric Denis, Rachid Mahalli, Alexis Delpierre, Christine Romagna, Denis Selimovic, Matthieu Renaud

**Affiliations:** 1 Department of Odontology, Tours University Hospital Center, 37000 Tours, France; rachid.mahalli@etu.univ-tours.fr (R.M.); delpierre.chir.dent@gmail.com (A.D.); christine.romagna@orange.fr (C.R.); drdenis.selimovic@gmail.com (D.S.); 2 Faculty of Dentistry, Nantes University, 44000 Nantes, France; 3 EA 75-05 Education, Ethics, Health, Faculty of Medicine, François-Rabelais University, 37000 Tours, France; 4 U1253, iBrain, CIC1415, Inserm, University Hospital Centre, Université de Tours, 37000 Tours, France; 5 Laboratory Bioengineering Nanosciences LBN, University of Montpellier, 34193 Montpellier, France; matt.renaud18@live.fr

**Keywords:** public health, psychobiological, health promotion, global health, psychological well-being

## Abstract

Psychobiological research is a systems approach that aims to integrate the biological, psychological and social systems that may influence health or pathology, particularly in chronic diseases and physical and/or psychiatric disorders. In this approach, we can expect to be able to deduce a ‘biological signature’ associated with particular symptom clusters. Similarly, psychosocial factors such as life events, health attitudes and behaviours, social support, psychological well-being, spirituality and personality are to be considered in terms of their influence on individual vulnerability to disease. At the psychophysiological level, it is important to understand, for example, the pathways that link the effects of chronic stress, social support and health, through the neuroendocrine and autonomic mechanisms that determine stress responses. At the macroscopic level, the role of individual socio-demographic variables such as personality, treatment modalities and health promotion through psycho-educational interventions needs to be explored.

## 1. Introduction

The biopsychological approach makes it possible to consider a person as a whole, not only physiologically but also in terms of psychological and biological factors, while taking into account the social environment in which he or she evolves. On a theoretical level, a set of explanatory hypotheses of health are considered on an equal footing, in a system of complex, multiple and circular causalities aimed at explaining a person’s behaviour [1].

It is difficult to date the birth of biopsychology precisely, although the “cell assembly theory” or “Hebb’s law” of the 1950s, which aimed to explain complex psychological phenomena such as perception, emotion, thought and memory through brain activity, marks the anchor of this scientific approach. This school of thought played a key role in the emergence of research in biopsychology [2]. According to Hebb’s law, learning is based on the consolidation of pre-existing synapses, determined by biological and genetic variables. One of the most recent applications of this law is related to mirror neurons, which are activated both when we perform a behaviour and when we see another living being doing the same thing. The theory of mirror neurons is currently accepted as the basis for explaining empathy [3].

In order to understand the biopsychosocial model, it is necessary to consider the transformation of the patient-object of the biomedical model, governed by stereotyped biological, physiological and mechanical balances, whose deficiencies generate pathology, into a patient-actor of his or her health, whose determinants are multiple [4]. From this perspective, the conditions for maintaining or improving health cannot be reduced to trying to modify individual behaviour without taking into account the social reality in which individuals live and from which they constantly receive and process information [5].

Observing these interconnections and describing them with extremely diverse methodological approaches based on human or non-human subjects and taking or not the form of fundamental or applied experiments, sets the framework and the stakes of the research of the biopsychological approach.

## 2. Complex Causal Ecosystems

It is now recognised that brain development and functioning is far from being entirely determined by the genetic programme. It is largely influenced by relational, emotional and environmental factors [6]. For example, early emotional deprivation induces severe and irreversible disturbances in brain development, resulting in various behavioural alterations [7,8]. This is why cognitive and emotional processes, including the subject’s interpretation of symptoms, and not psychopathological aspects are largely considered in this model [4]. This involves an often-uncomfortable navigation for clinicians, patients and researchers between the complexity and uncertainty of being able to explain psychological functioning by physiology and chemistry. This “global” approach opens up avenues of research to try to deduce a “biological signature” associated with particular symptom clusters. This context imposes a broadening of research perspectives in multiple, varied and interconnected fields but also with the active participation of the patient, whose perspective on the perception of his or her health condition is essential.

In trying to understand this complex causal ecosystem, it is important to look at it from multiple angles without forgetting some basic elements. For example, people from lower socio-economic groups have an almost universal tendency to die younger and to be sicker during their lifetime in terms of frequency [9]. Some people die at a very early age, others suffer from chronic diseases or disabilities and some live to a very old age. These differences can be analysed by region, race, gender or age, as well as by indicators of socio-economic status [10,11,12]. All these factors reveal systematic patterns in the distribution of health, both between and within societies, so that not everyone is born with the same chances of living a long and healthy life. There are also recurrent elements in the individual’s environment that increase the risk of disease. These include environmental risk factors such as chemicals, combustion emissions, radioactivity, industrial waste, accidents at home, at work or in traffic, etc. There are also factors such as the apnoea-hypopnoea index, family and social support or socio-economic status that could favour the development of certain types of diseases such as anxiety disorders or cancers [13,14]. Finally, heredity, smoking, metabolic diseases such as diabetes, obesity, hypertension, hypercholesterolemia, hyperglycemia, sedentary lifestyle and stress are potential mediators with a measurable impact on disease processes and should not be considered as confounding factors in studies [15,16,17,18,19].

## 3. Many Unexplored Sub-Systems

In recent years, high-throughput sequencing techniques have highlighted the complex influence of the gut and/or oral flora on host metabolism, nutrition and immune function. For example, alteration in the microbiota or dysbiosis plays a central role in functional bowel disorders [20]. Dysbiosis has been particularly studied in irritable bowel syndrome, and studies in animals have shown that this imbalance is involved in the visceral hypersensitivity observed, as well as in intestinal motor disorders [21]. Finally, dysbiosis is thought to favour alteration in the intestinal barrier by increasing intestinal permeability. This would facilitate the passage of bacterial antigens at the origin of a low-grade inflammation, leading to the sensitisation of the sensory afferents of the enteric nervous system [22].

Further work has shown that dysbiosis is a condition associated not only with gastrointestinal disorders but also with diseases affecting other organs. The nervous system and the gastrointestinal tract communicate through a bidirectional network of signalling pathways called the gut–brain axis, which consists of multiple connections including the vagus nerve, the immune system, metabolites and bacteria [22,23]. Other studies also suggest potential effects of the oral microbiota on biochemical signalling events between the oral microbiota and the central nervous system [24].

During dysbiosis, these pathways are regulated and associated with altered blood–brain barrier permeability and neuroinflammation. Thus, many mechanisms underlie the impact of the gut microbiota on neurodevelopment. Among these, the inflammasome pathway has been associated with neuroinflammatory diseases such as multiple sclerosis, Alzheimer’s disease and Parkinson’s disease but also with anxiety and depressive disorders [25]. The literature in this approach is abundant, but the contradictory results in terms of bacterial composition depending on the studies and the methodologies employed do not yet allow the use of the microbiota and its metabolites as a relevant marker for diagnosis, monitoring of disease progression or response to treatment. However, the literature confirms the importance of the potential impact of approaches linked to modulations of intestinal and oral bacterial populations. These approaches are all the more important, as micro-organisms are an essential component of the ecosystem and have a significant impact on the environment in which we live.

## 4. Towards an Active Participation of the Patient

The diagnosis and announcement of a serious illness is a major life event that influences individual functioning, emotional well-being and challenges the doctor–patient relationship. For many patients, the years following diagnosis are particularly difficult in terms of coping with the disease. Altered quality of life and social functioning can lead to psychological symptoms, such as distress, anxiety and depression [26]. Various areas of life, such as education, work, relationships and social participation, need to be adjusted in order to gain a new sense of coherence that must necessarily include the disease [26]. Furthermore, patients’ adaptation to illness shows a wide range of possible reactions to the diagnosis and different levels of resilience. According to Southwick et al., “the determinants of resilience include a multitude of biological, psychological, social and cultural factors that interact with each other to determine how an individual responds to stressful experiences” [27]. Caregivers must always keep in mind that the determinants of health and disease are multiple and diverse and that the therapeutic strategies envisaged will include, in addition to the “traditional” modalities aimed at modifying physiological parameters, various means of acting on the psychosocial factors perceived as participating in the health problem insofar as the patient’s beliefs and expectations directly influence the results of the treatments [28]. Consequently, discrepancies between lay and scientific representations of the disease need to be discussed: the corollary of active patient participation is a particular emphasis on education and information [28,29]. However, it is necessary to clarify what is meant by “education”: it is certainly not a lecture that one hopes will “correct cognitive distortions”. Rather, it is a didactic process in which the patient’s beliefs are tested against the facts and thus gradually adapted [30]. We need to embark on a process of change in relation to the therapeutic relationship, which requires the development of a wider range of relational and educational skills. This requires a rethinking of the carer–patient relationship, drawing on broader theoretical knowledge and interpersonal skills in order to meet the patient in all his or her uniqueness, as suggested by the biopsychosocial approach.

## 5. Health-Related Quality of Life and the Biopsychosocial Model

The extension of health-relevant parameters to the biopsychosocial domain poses problems of assessment and especially of quantification of subjective values. Health-related quality of life (HRQoL) as an indicator started to be used extensively from the 1980s onwards, especially in the management of patients with chronic diseases. A patient’s health and ability to function depend on and are the consequence of several components: physical, mental and social. Therefore, in a biopsychosocial approach, patients need to be assessed holistically, and the functioning of all three components thus needs to be taken into account for a patient to progress as a person and as a social individual [31,32]. In this context, quality of life estimates and measures the living conditions of patients that are related to health status and/or disease dependency or response when evaluating the success of therapeutic or surgical goals in the medical field [32,33]. In other words, the HRQoL indicator is currently a valuable tool for understanding circumstances related to illness and medical care. However, some of these tools are open to criticism, especially with regard to the method used to develop the measurement scales, as they often do not take into account the patients’ perspective as a whole, their perception and social representations of the disease. Finally, they do not always take into account the evolution of medical knowledge and therapeutics, which sometimes disrupt perceptions and living conditions. Furthermore, there are discrepancies between the perception of patients’ quality of life and that of health experts. These discrepancies sometimes invalidate measurement tools (if they are not designed from the patients’ perspective) and may even contribute to a decrease (or non-improvement) in patients’ quality of life due to inadequate therapeutic practices [34]. It is important to assess the perceived quality of life of patients using quality of life scales that take into account the perspective and “subjectivity” of patients in their living whole (as opposed to experimental observation) in relation to the disease specifically. This means taking into account factors such as coping with the disease, denial of the disease and temporal factors, as well as all the dimensions of quality of life suggested by the patients and the various members of the healthcare team [35]. Determining the dimensions of the quality of life perceived by the subjects studied amounts to attributing the factors that explain a decrease or an improvement in the quality of life as a whole with conceptually reliable and psychometrically rigorously validated measurement scales [36].

## 6. Conclusions

Despite its limitations, and insofar as the number of parameters taken into account is necessarily limited in relation to the number and variety of health determinants, which are almost infinite, the biopsychosocial model is the most successful theoretical and clinical model of health and illness that we currently have.

In order to go further within the framework of this model, to better care for the patients who call on us, to better understand all the components of who they are, to shake up our beliefs and achievements and to feed ourselves with the production of knowledge made possible by the biopsychosocial approach, this Special Issue is aimed at a wide audience of researchers who can contribute to the emergence of new knowledge in this field.
